# Repurposing Rafoxanide: From Parasite Killer to Cancer Fighter

**DOI:** 10.3390/biomedicines13071686

**Published:** 2025-07-09

**Authors:** Teresa Pacifico, Lorenzo Tomassini, Livia Biancone, Giovanni Monteleone, Carmine Stolfi, Federica Laudisi

**Affiliations:** 1 Department of Systems Medicine, University of Rome “Tor Vergata”, 00133 Rome, Italy; teresa.pacifico@uniroma2.it (T.P.); lorenzo.tomassini19@gmail.com (L.T.); biancone@med.uniroma2.it (L.B.); gi.monteleone@med.uniroma2.it (G.M.); carmine.stolfi@uniroma2.it (C.S.); 2 Gastroenterology Unit, Policlinico Universitario Tor Vergata, 00133 Rome, Italy

**Keywords:** colorectal cancer, anthelmintic drug, drug repurposing, drug repositioning, halogenated salicylanilide, niclosamide, STAT3, NF-κB, endoplasmic reticulum stress, immunogenic cell death

## Abstract

Rafoxanide, originally developed as a veterinary anthelmintic for the treatment of parasitic infections in livestock, has recently emerged as a promising therapeutic prospect in oncology. This compound has demonstrated notable antineoplastic effects against a variety of cancers, including skin, gastric, colorectal, and lung cancers, as well as hematological malignancies such as multiple myeloma. Rafoxanide exerts its anticancer activity through multiple complementary mechanisms, including the induction of endoplasmic reticulum stress, cell cycle arrest, apoptosis, and immunogenic cell death. Furthermore, the drug has been reported to inhibit key oncogenic signaling pathways (e.g., STAT3, NF-κB, c-FLIP, survivin) that contribute to tumor growth and metastasis. Preclinical studies in murine models have demonstrated significant reductions in tumor volume of up to 50% and a tumor-free rate exceeding 80%, with effective doses ranging from 7.5 to 40 mg/kg. This multitargeted mode of action distinguishes rafoxanide from conventional therapies and may help overcome resistance mechanisms that often limit the efficacy of cancer treatments. In this review, we summarize and discuss the growing body of evidence supporting rafoxanide’s therapeutic potential in oncology, as well as its possible applications in cancer treatment.

## 1. Introduction

Rafoxanide is an anthelmintic compound developed in 1969 to treat parasitic infections in livestock, particularly ruminants like cattle and sheep. The molecule has received Food and Drug Administration (FDA) approval for the treatment of fascioliasis caused by the trematode parasite *Fasciola hepatica*, which can cause significant economic losses in agriculture due to its harmful effects on livestock health [1].

Rafoxanide exhibits a broad spectrum of anthelmintic activity, targeting gastrointestinal nematodes and parasites such as *Haemonchus* spp. and *Oestrus ovis*. Its mechanism of action is based on mitochondrial uncoupling, which disrupts parasite mitochondrial function by interfering with oxidative phosphorylation and altering proton gradients [2,3,4].

**Figure 1:**
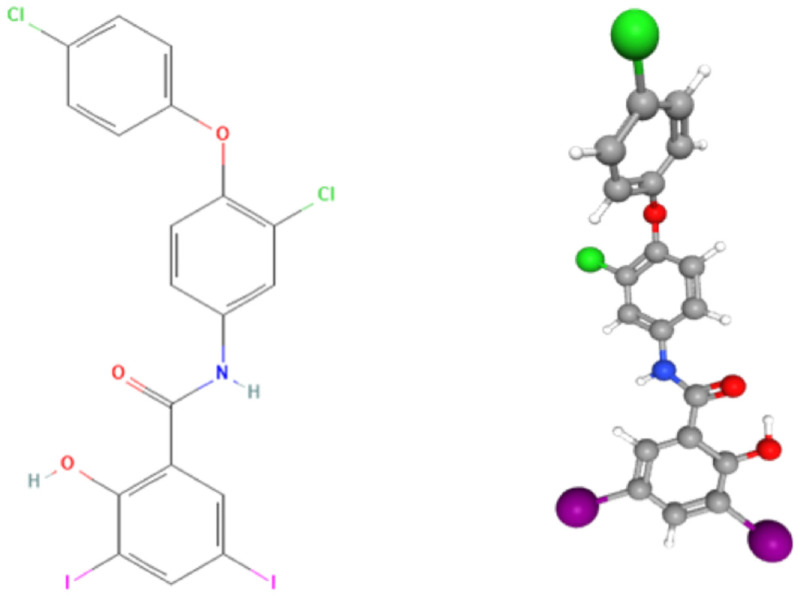
Chemical structure of rafoxanide. The color scheme used in the 2D structural formula on the left (e.g., green for chlorine, red for oxygen, blue for nitrogen, purple for iodine) is maintained in the 3D molecular model on the right to allow direct comparison of corresponding atoms. Sources: PubChem [Internet]. Bethesda (MD): National Library of Medicine (US), National Center for Biotechnology Information; 2004-. PubChem Compound Summary for CID 31475, Rafoxanide. Available from: https://pubchem.ncbi.nlm.nih.gov/compound/Rafoxanide (accessed on 30 June 2025).

From a chemical standpoint, rafoxanide is a halogenated salicylanilide compound with a molecular weight of 626.01 g/mol and a melting point of 168–170 °C (Figure 1). It appears as a greyish-white crystalline powder, with a molecular formula of C_19_H_11_Cl_2_I_2_NO_3_, corresponding to 3′-chloro-4′-(p-chlorophenoxy)-3,5-diiodosalicylanilide. Rafoxanide belongs to a family of weakly acidic phenolic agents, characterized by a salicylic acid core linked to an anilide moiety, typically featuring a lipophilic substituent, such as a tert-butyl group at the 3-position [5]. An aryl chain within the aniline portion of the molecule further contributes to its overall stability and biological activity. The 3,5-diiodosalicyloyol group has been identified as the pharmacologically active component crucial for its therapeutic effects [6].

Due to its chemical nature, rafoxanide is moderately soluble in organic solvents such as acetone, chloroform, ethyl acetate, acetonitrile, and methanol, while remaining insoluble in water [7]. The synthesis of rafoxanide has undergone significant optimization to improve efficiency and reduce the use of hazardous reagents [6,8,9]. Earlier synthesis methods relied on iodine chloride (ICl) for the iodination step, which posed challenges due to its instability, toxicity, and difficulty in handling, leading to low yields. Later approaches explored the use of preformed 3,5-diiodosalicylic acid, but the production of this intermediate still involved ICl or other methods with poor atom economy [7]. More recently, a streamlined synthetic approach has been developed, reducing the process to three main steps and achieving a total yield of 74% [10]. This improved method utilizes iodine and hydrogen peroxide for efficient salicylic acid iodination, achieving yields of up to 95%. Furthermore, it employs a one-pot condensation reaction involving acid chloride, bypassing intermediate purification steps and increasing the final product yield to 82%. These advancements have made the production of rafoxanide and its analogues more cost-effective and environmentally sustainable [10].

Despite extensive chemical and synthetic characterization, the available literature provides limited data on rafoxanide’s pharmacokinetics (PK), bioavailability, formulation strategies, and drug delivery systems, particularly beyond its veterinary applications. Most existing PK studies have been conducted in ruminants, where oral administration results in a prolonged absorption phase, with a lag time of approximately 5 h and a Tmax occurring around 5 h post-dosing [11,12]. Bioavailability appears to be highly dependent on formulation: modified and generic oral suspensions in sheep show 35–45% lower systemic exposure (AUC) compared to innovator products, highlighting how formulation significantly influences pharmacokinetic profiles [13]. Furthermore, intra-abomasal administration results in peak plasma concentrations up to 3.5 times higher than intraruminal delivery, demonstrating that the administration route greatly impacts absorption efficiency [14,15].

Rafoxanide’s physicochemical properties—namely its high lipophilicity and poor aqueous solubility—pose significant challenges for effective drug delivery. To address this limitation, polymeric complexation strategies—such as those involving povidone—have been shown to improve solubility through hydrogen bonding and π–π stacking interactions, offering potential routes to improved oral bioavailability [12,16,17]. Approved veterinary formulations currently employ carriers such as carboxymethyl cellulose and Tween-80. Furthermore, co-administration studies show pharmacokinetic interactions, such as higher plasma levels of ivermectin when given with rafoxanide, highlighting the need for comprehensive studies of PK and drug interaction, especially in multi-drug regimens [11]. In particular, pharmacokinetic, biodistribution, metabolism, and advanced drug delivery studies focused on humans are still lacking, which means rafoxanide is not currently approved for human use.

## 2. Rafoxanide and Cancer Treatment

Drug repurposing, also known as drug repositioning, is a strategic approach to broadening the use of established medications [18,19,20]. This method helps to bypass the extensive regulatory requirements typically associated with the development of new drugs. Using existing data on absorption, distribution, metabolism, excretion, and toxicity (ADMET), drug repurposing accelerates the development timeline and reduces costs, especially for compounds that have already passed Phase III clinical trials, demonstrating their safety and efficacy in various patient populations [21,22]. This approach not only improves the drug development pipeline but also maximizes the therapeutic potential of existing medications for the treatment of a variety of diseases. The field has transformed into a data-driven science, utilizing bioinformatics and cheminformatics to uncover connections between biological entities and pharmacological compounds.

**Figure 2:**
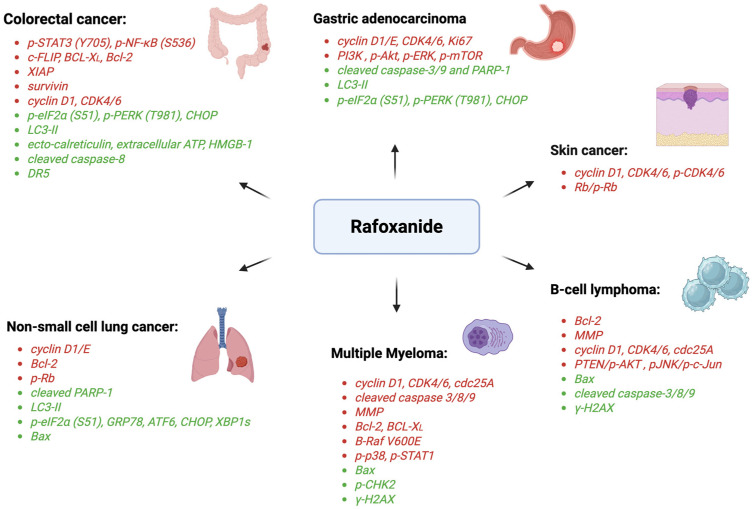
Molecular targets of rafoxanide in different cancer types. Abbreviations: p-STAT3, phosphorylated signal transducer and activator of transcription 3; p-NF-κB, phosphorylated nuclear factor kappa-light-chain-enhancer of activated B cells; c-FLIP, cellular FLICE-like inhibitory protein; BCL-XL, B-cell lymphoma-extra large; Bcl-2, B-cell lymphoma/leukemia-2; XIAP, X-linked inhibitor of apoptosis; CDK 4/6, cyclin-dependent kinase 4/6; p-EIF2α, phosphorylated eukaryotic initiation factor 2α; p-PERK, phosphorylated PKR-like endoplasmic reticulum kinase; CHOP, C/EBP homologous protein; ATP, adenosine triphosphate; HMGB1, high mobility group box 1; DR5, death receptor 5; p-Rb, retinoblastoma protein; PARP-1, Poly(ADP-ribose) polymerase-1; ATF6, activating transcription factor 6; Xbp1s, spliced form of X-box binding protein 1; BAX, Bcl-2-associated X protein; PI3K, phosphatidylinositol 3-kinase; p-mTOR, phosphorylated mammalian target of rapamycin; p-ERK, phosphorylated extracellular signal-regulated kinase; MMP, mitochondria membrane potential; B-Raf, B-rapidly accelerated fibrosarcoma; p-CHK2, phosphorylated checkpoint kinase 2; γ-H2AX, γ-H2A histone family member X; PTEN, phosphatase and tensin homolog; p-JNK, phosphorylated Jun N-terminal kinase. Created with Biorender.com.

In this context, recent studies have shown that rafoxanide has additional functions other than the anthelmintic effects [23,24,25,26,27], and an accumulating body of evidence suggests that rafoxanide possesses significant anticancer properties. Here, we review and discuss the experimental findings on the ability of rafoxanide to combat malignant cell growth in different cancer types (Table 1, Figure 2).

### 2.1. Skin Cancer

Skin cancer represents a heterogeneous group of malignancies and constitutes a significant global health concern, effectively qualifying it as a worldwide epidemic. Skin cancer is categorized primarily into two main types: melanoma skin cancer (MSC) and non-melanoma skin cancers (NMSCs), with the latter including squamous cell carcinoma (SCC) and basal cell carcinoma (BCC) [37]. Collectively, these categories account for more than 90% of all skin cancer cases. It should be noted that one in three cancers diagnosed globally is skin cancer, with NMSCs being the variant that occurs most frequently [37].

A key feature of skin cancer development is abnormal regulation of the cell cycle and cellular proliferation [38]. Cyclin-dependent kinases 4 and 6 (CDK4/6) play a crucial role in the transition of cells from the G1 phase to the S phase of the cell cycle. Dysregulation of these kinases has been linked to cancer progression, with studies showing that CDK4/6 are overexpressed in more than 90% of skin cancer cases [39,40].

A study by Shi et al. investigated the potential of repurposing existing drugs as dual inhibitors of CDK4/6 [28]. Using advanced virtual screening techniques, the authors examined a library of FDA-approved small molecules to find candidates that might inhibit CDK4/6 activity. In fact, current CDK4/6 inhibitors such as palbociclib, ribociclib, and abemaciclib are mainly approved for breast cancer treatment, but come with limitations such as side effects and the requirement for an intact retinoblastoma protein [41,42]. Rafoxanide was found to function as a dual CDK4/6 inhibitor in human skin cancer cell lines, demonstrating significant cytotoxic effects against A375 and A431 cells, which were characterized by cell cycle arrest in the G1 phase and reduced expression/activity of CDK4/6, cyclin D, and retinoblastoma proteins [28]. In particular, rafoxanide exhibited the highest cytotoxic activity with IC_50_ values of 1.09 µL for A375 and 1.31 µL for A431 cells. In vivo studies using BALB/c nude mice xenografted with A357 cells showed that intraperitoneal injection of rafoxanide with a dose of 40 mg/kg led to significant antitumor effects, evidenced by a notable reduction in tumor growth rates that were comparable to those seen with oxaliplatin (5 mg/kg), a standard chemotherapeutic agent used in first-line treatment protocols [28]. Furthermore, combined administration of rafoxanide and oxaliplatin resulted in a synergistic enhancement of therapeutic response, highlighting the potential for combination therapy strategies [28]. However, the effect of rafoxanide on CDK4/6 expression was not further investigated in this model.

These results suggest that rafoxanide may be a promising candidate for future preclinical and clinical studies, either as a single treatment or in combination with established chemotherapy drugs to treat skin cancer.

### 2.2. Gastric Adenocarcinoma

Gastric adenocarcinoma, which accounts for 90–95% of all gastric cancer cases, is a highly lethal malignancy that often exhibits resistance to treatment, making the search for alternative therapeutic options a critical priority [43,44,45]. Liu et al. have demonstrated rafoxanide’s antineoplastic effects in gastric adenocarcinoma cell lines, showing a remarkable ability to inhibit cell proliferation while promoting both apoptotic and autophagic processes [29]. In fact, in vitro assays conducted on SGC-7901 and BGC-823 cells showed that rafoxanide decreased cell viability in a dose- and time-dependent manner, while exhibiting minimal cytotoxic effects in normal gastric epithelial cells (i.e., GES-1) [29]. Flow cytometry analyses indicated that rafoxanide treatment caused cell cycle arrest in the G0/G1 phase, which was associated with a significant downregulation of cyclins D1 and E [29]. Mechanistic studies revealed that rafoxanide activates the mitochondrial apoptotic pathway, as evidenced by a loss of mitochondrial membrane potential (MMP), upregulation of cleaved caspase-3 and caspase-9, and the release of cytochrome c into the cytosol [29]. Additionally, PARP-1 cleavage was observed, while caspase-8 activity remained unchanged [29], indicating that rafoxanide’s pro-apoptotic effects are mediated through intrinsic apoptotic mechanisms rather than extrinsic pathways. The study also demonstrated that rafoxanide could trigger autophagy, evidenced by increased LC3-II protein expression, the formation of autophagic vacuoles, and enhanced autophagic flux [29]. Pre-treatment with 3-methyladenine, which blocks autophagosome formation, inhibited the accumulation of LC3-II and apoptosis, indicating that autophagy plays a significant role in the overall mechanisms of cell death in gastric adenocarcinoma cells. Further investigation showed that rafoxanide has antitumor effects by suppressing the PI3K/Akt/mTOR signaling pathway, which is crucial for cancer cell survival [29]. This suppression occurred through downregulation of Akt and mTOR phosphorylation [29]. Importantly, activating this pathway with the PI3K agonist 740Y-P could reverse both the pro-apoptotic and autophagic effects triggered by the drug, thus highlighting the critical role of the PI3K/Akt/mTOR cascade in the rafoxanide-driven anticancer effects [29]. To confirm these in vitro findings, in vivo studies were carried out using an animal xenograft model, in which mice received rafoxanide by oral administration [29]. The results revealed a significant decrease in tumor volume with no notable toxicity compared to the control group [29]. Immunohistochemical analysis of tumor tissues revealed elevated expression of apoptotic and autophagic markers, accompanied by a decrease in cell proliferation and Akt phosphorylation levels [29]. Furthermore, transmission electron microscopy further validated these findings, showing a higher presence of autophagic vacuoles in tumor tissues from mice treated with rafoxanide [29].

Collectively, this study demonstrates that rafoxanide effectively limits the growth of gastric adenocarcinoma cells both in vitro and in vivo by modulating the PI3K/Akt/mTOR pathway, resulting in autophagy and apoptosis. Its ability to induce these two molecular processes highlights the need to further explore rafoxanide as a repurposed anticancer treatment.

### 2.3. Colorectal Cancer

Colorectal cancer (CRC) remains one of the leading causes of cancer-related deaths worldwide, mainly due to the lack of effective treatment for advanced disease [46]. In addition to surgical intervention, immunotherapy and chemotherapy are the mainstays of CRC treatment. Unfortunately, the intrinsic and/or acquired resistance of CRC cells to these strategies, the presence of redundancy mechanisms limiting the efficacy of immunotherapeutics, and the severe side effects of commonly used cytostatics represent major drawbacks [47].

A significant challenge in the treatment of CRC is tumor heterogeneity, which makes standard therapeutic approaches less effective [38]. Furthermore, the immunosuppressive nature of the tumor microenvironment adds to therapeutic resistance and disease progression [48]. Recent preclinical research from 2019 to 2024 highlights the potential of rafoxanide as a therapeutic agent in CRC treatment. Studies have demonstrated its efficacy in vitro, ex vivo using patient-derived organ cultures and colon organoids, as well as in vivo models that mimic sporadic and inflammation-associated CRC [30]. Early research conducted in 2019 indicated that rafoxanide exhibits antiproliferative effects on several colorectal cancer cell lines, including HCT-116 and DLD1 [30]. Further exploration of the potential of rafoxanide for the treatment of CRC revealed its ability to inhibit the proliferation of cancerous cells, but not normal colonic epithelial cells (i.e., HCEC-1CT) [30]. These characteristics highlight the potential of rafoxanide as a potent and selective anticancer agent in CRC treatment. Rafoxanide’s mechanism of action involves a marked reduction in cyclin D1 protein levels, leading to cell cycle arrest at the G0/G1 phase, and the activation of a caspase-dependent apoptotic pathway [30]. Notably, the reduction in cell proliferation was reversible upon drug withdrawal, suggesting a cytostatic rather than a purely cytotoxic effect [30]. Further investigation into the underlying mechanisms has shown that the effects induced by rafoxanide occur primarily through endoplasmic reticulum stress (ERS). This is demonstrated by increased phosphorylation of eukaryotic initiation factor 2α (eIF2α) and induced transcription of C/EBP homologous protein (CHOP) [30]. The connection between ERS induction and reduced cell proliferation was supported using tauroursodeoxycholic acid (TUDCA), a chaperone recognized for its ability to alleviate ERS. TUDCA effectively inhibited rafoxanide-induced eIF2α phosphorylation, along with the downregulation of cyclin D1 protein and cell cycle arrest in the G0/G1 phase [30]. Beyond its growth-inhibiting effects, rafoxanide also showed pro-apoptotic activity in CRC cell lines, which depended on caspase activation [30]. Of note, the use of the pan-caspase inhibitor Q-VD-Oph completely blocked rafoxanide-induced cell death [30]. In human CRC explants, rafoxanide treatment led to a significant reduction in Ki-67 positive proliferating cancer cells while sparing normal colon proliferating cells [30].

Finally, in vivo observations in *Apc*^min/+^ mice, which spontaneously develop intestinal lesions, indicate that systemic administration of rafoxanide markedly decreased both the number and the size of colonic lesions [30]. Immunohistochemical analyses corroborated these findings by confirming decreased cellular proliferation and increased apoptosis specifically within neoplastic tissues [30].

**Figure 3:**
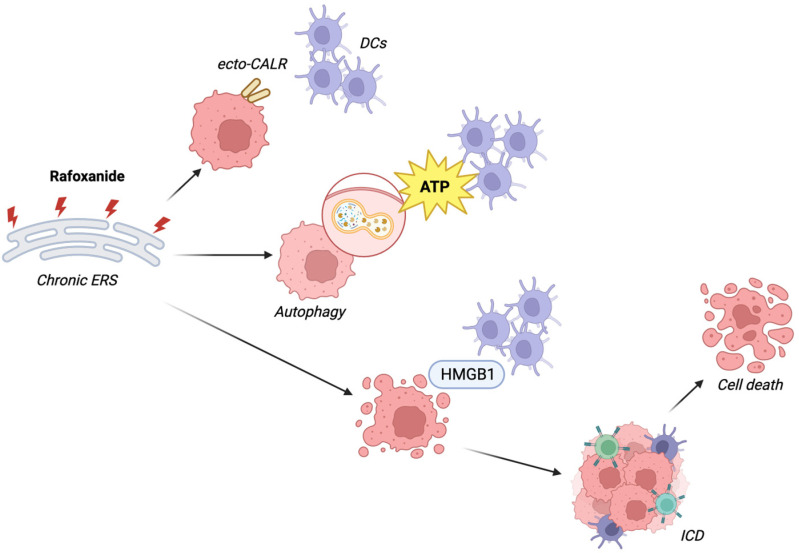
Effect of rafoxanide on ICD-related markers. Abbreviations: ERS: endoplasmic reticulum stress; CALR: calreticulin; DCs, dendritic cells; ATP, adenosine triphosphate; HMGB1, high mobility group box 1; ICD, immunogenic cell death. Created with Biorender.com.

Building on the findings that identified rafoxanide as an inducer of ERS, subsequent studies have validated its ability to trigger immunogenic cell death (ICD) in CRC cells [31]. This process, a form of apoptosis associated with ERS induction, involves the expression and release of specific damage-associated molecular patterns (DAMPs). These include surface exposure of calreticulin (CALR), release of adenosine triphosphate (ATP), and secretion of the high mobility group box 1 (HMGB1), all of which contribute to the activation of antitumor immune responses [49]. Evidence from both murine CRC models and human studies has demonstrated that rafoxanide is capable of inducing all three key markers of ICD (Figure 3) [31]. Additionally, vaccination trials with syngeneic CT26 cells pre-treated with rafoxanide demonstrated significant inhibition of tumor growth (75% with no visible sign of tumor growth and 25% with only tiny masses ranging between 6 and 13.5 mm^3^) and increased tumor-free survival in immunocompetent mice [31].

Altogether, these findings suggest that rafoxanide may act through a dual mechanism: directly inducing cell death in neoplastic cells while simultaneously activating the immune system. This supports the rationale for future combination therapies involving immunotherapeutic agents.

Further mechanistic insights revealed that rafoxanide could sensitize CRC cells to TRAIL (TNF-related apoptosis-inducing ligand)-based treatments [32]. TRAIL, also known as CD253, is a protein within the TNF superfamily that triggers programmed cell death by engaging with death receptors (DR) [50]. Five human TRAIL receptors have been identified (DR4, DR5, DcR1, DcR2, and osteoprotegerin). Two of these, DR4 (TRAIL-R1) and DR5 (TRAIL-R2), contain cytoplasmic death domains and activate apoptotic pathways upon TRAIL binding [51]. Decoy receptors DcR1 (TRAIL-R3) and DcR2 (TRAIL-R4) are also expressed on the cell surface but lack functional intracellular death domains; they compete with DR4/DR5 for TRAIL binding, thereby conferring protection against TRAIL-induced apoptosis [51]. TRAIL can also bind to a soluble, low-affinity receptor known as osteoprotegerin, although its physiological relevance remains unclear [52]. Interaction of TRAIL with DR4 and DR5 activates caspase-8 through the death-inducing signaling complex (DISC), which results in apoptosis. TRAIL selectively targets cancerous cells, as normal cells exhibit higher expression of DcRs, positioning it as a promising candidate for cancer therapy. However, many types of cancer, including CRC, develop resistance to TRAIL-induced cell death, limiting its clinical utility [53,54,55]. At the molecular level, rafoxanide is a specific TRAIL sensitizing agent that makes CRC more vulnerable to TRAIL-induced apoptosis while sparing normal colon epithelial cells [32]. In particular, it increases the expression of DR5 in CRC cells at both transcript and protein levels, thus increasing the cell capacity to detect and respond to TRAIL signals [32]. Notably, other death and decoy receptors (DR4, DcR1, DcR2) remained basically unchanged [32]. Rafoxanide promotes caspase-8 activation within cells and downregulates c-FLIP and survivin [32], two vital anti-apoptotic proteins which are typically responsible for resistance to TRAIL therapy [56]. This downregulation occurs through post-translational modifications rather than changes in gene expression. In fact, the decrease in c-FLIP and survivin is associated with proteasome-mediated degradation, which enhances TRAIL sensitivity in previously resistant cells in a dose-dependent manner [32]. Additionally, the proteasome inhibitor lactacystin nearly completely counteracts the decrease in survivin levels caused by rafoxanide, further supporting this mechanism. Silencing both c-FLIP and survivin in CRC cells using specific antisense oligonucleotides triggers an apoptotic response similar to that of combinatory treatment, underscoring the importance of these targets in the sensitizing effect of rafoxanide [32]. These results were confirmed using patient-derived CRC organoids and in vivo CT26 graft mouse models, showing enhanced tumor inhibition and increased apoptosis when rafoxanide was paired with TRAIL [32]. Notably, this combination treatment was well tolerated, with no significant changes in body weight observed in treated mice compared to the control group [32].

Our group has recently revealed the potential of rafoxanide to treat colitis-associated cancer (CAC), which develops in the context of chronic inflammatory bowel diseases [33]. In particular, rafoxanide exhibits remarkable anticancer potential by serving as an effective suppressor of the activation of STAT3 and NF-κB, two transcription factors playing a central role in the development of different types of CRC, in the CAC and sporadic CRC microenvironment [57]. In particular, experimental studies in sporadic (*Apc*^min/+^ mice) and inflammation-associated (azoxymethane/dextran sulfate sodium-driven) preclinical mouse models of CRC showed that rafoxanide treatment resulted in a significant reduction in both the number and size of colonic tumors while sparing normal intestinal mucosa [33]. The antitumor effects of rafoxanide were linked to a significant reduction in the activation of STAT3 and NF-κB in both epithelial and immune cell compartments [33]. This inhibitory action on inflammatory signaling affects the ability to produce pro-tumorigenic cytokines, resulting in reduced levels of IL-6 and TNF-α, while increasing the frequency of CD3^+^ secreting IFN-γ [33]. The ability of rafoxanide to affect the tumor microenvironment introduces a unique mechanism of action that complements its direct effects on malignant cells. In fact, by targeting both cancer cells and the inflammatory microenvironment that supports their growth, rafoxanide may address multiple facets of CAC pathogenesis, offering a comprehensive therapeutic strategy.

### 2.4. Non-Small Cell Lung Cancer

Non-small cell lung cancer (NSCLC) presents a great challenge to global health, driving the need for novel therapeutic interventions [58,59]. In a recent study by Hu et al., rafoxanide was shown to significantly inhibit the proliferation, invasion, and migration of NSCLC cells in vitro in a dose-dependent manner [34]. Treatment resulted in apoptotic cell death, underscoring its potential efficacy in the clinical setting [34]. Moreover, RNA sequencing analysis indicated that rafoxanide activates a suite of genes related to ERS, suggesting a mechanistic interplay between this molecular pathway and the anti-cancer properties of the drug [34]. The induction of ERS after rafoxanide treatment initiated the unfolded protein response (UPR), a cellular mechanism aimed at restoring homeostasis under stressed conditions. Although autophagy was activated as a protective response to alleviate ERS, the sustained or excessive nature of this stress ultimately triggers apoptosis in NSCLC cells [34]. In vivo experiments using mouse models xenografted with A549 cells confirmed that rafoxanide acts as an antitumor drug against NSCLC [34]. Mice that received rafoxanide at a dosage of 15 mg/kg over a 14-day period demonstrated a statistically significant reduction in tumor volume growth rates compared to the control group [34]. Ki67 immunostaining assays indicated that rafoxanide inhibits tumor cell proliferation in vivo [34]. In particular, treatment did not appear to induce significant systemic toxicity in these animal models, as evidenced by the absence of substantial differences in body weight measurements between the rafoxanide-treated and control groups, suggesting that rafoxanide has an acceptable safety profile [34]. Collectively, these in vivo findings propose that rafoxanide effectively suppresses the growth of NSCLC tumors while exhibiting minimal adverse effects. Furthermore, comprehensive biochemical assessments appear to support the in vivo safety of rafoxanide as mice given doses from 10 to 30 mg/kg did not show significant changes in hepatic biomarkers (ALT, ALB, AST) or renal function markers (BUN, CREAT) compared to untreated controls.

### 2.5. Multiple Myeloma

Multiple myeloma (MM) is a type of hematologic cancer characterized by clonal growth of malignant plasma cells in the bone marrow. This growth is often supported by a microenvironment that hinders the effectiveness of standard treatments [60]. Despite therapeutic advances, including proteasome inhibitors and immunomodulatory agents, MM remains incurable [61,62]. In this context, rafoxanide has garnered interest as a potential novel treatment that may overcome resistance presented by the microenvironment, due to its unique biological properties. Notably, rafoxanide has been identified as an inhibitor of B-Raf, a serine/threonine kinase whose mutations are implicated in the pathogenesis of several cancers [63]. Li and co-workers reported that rafoxanide has a significant antitumor effect on MM cells, both in vitro and in vivo [35]. In particular, rafoxanide demonstrated a dose- and time-dependent inhibitory effect on the proliferation of various MM cell lines, including ARP1 and OCI-MY5 cells, as well as on patient-derived CD138^+^ MM cells [35]. Rafoxanide exhibited dose-dependent cytotoxic effects, with IC_50_ values ranging from 19.2 to 47.2 μM across various multiple myeloma cell lines, specifically 19.2 μM in H929, 40.1 μM in H929R, and 27.8 μM in OCI-MY5 cells. These cytotoxic effects were maintained even in the presence of bone marrow stromal cells (BMSCs) and survival-promoting cytokines such as IL-6 and IGF-1, suggesting that rafoxanide can overcome microenvironment-mediated drug resistance [35]. Moreover, it selectively induced apoptosis in MM cells through both intrinsic and extrinsic pathways, as evidenced by decreased mitochondrial membrane potential, activation of caspase-3, -8, and -9, downregulation of anti-apoptotic proteins (Bcl-2, Bcl-XL), and upregulation of the pro-apoptotic protein Bax [35]. Furthermore, rafoxanide triggered cell cycle arrest at the G0/G1 phase by downregulating key cell cycle regulators and enhancing DNA damage response signaling [35]. Additionally, rafoxanide induced cell cycle arrest at the G0/G1 phase, an effect associated with decreased expression of cyclin D1, CDK4, CDK6, and CDC25A, along with increased phosphorylation of CHK2 [35]. The drug also activated the DNA damage response, as evidenced by elevated levels of γ-H2AX [35]. Considering the established role of the MAPK pathway in the progression of multiple myeloma [64,65], the study also assessed the impact of rafoxanide on MAPK signaling. The results showed that rafoxanide inhibited the phosphorylation of p38 MAPK and STAT1 [35]. In vivo administration of rafoxanide every two days for 14 days significantly reduced tumor growth in an MM xenograft mouse model, without causing any apparent toxicity [35]. Immunohistochemical analysis confirmed decreased proliferation, increased apoptosis, enhanced DNA damage signaling, and inhibition of MAPK pathway activity in tumor tissue [35]. Finally, rafoxanide exhibited a synergistic cytotoxic effect when combined with the proteasome inhibitor bortezomib or the immunomodulatory agent lenalidomide, suggesting its potential value in combination therapy [35].

### 2.6. B-Cell Lymphoma

Diffuse large B-cell lymphoma (DLBCL) is an aggressive type of non-Hodgkin lymphoma, accounting for a significant proportion of newly diagnosed lymphoma cases [66]. Despite improvements with standard R-CHOP therapy—a combination of rituximab, cyclophosphamide, doxorubicin, vincristine (Oncovin), and prednisone—DLBCL continues to pose treatment challenges, with many patients developing refractory or relapsed disease [67]. In 2020, Wan and colleagues assessed the impact of rafoxanide on DLBCL cellular function [36]. The results indicated that rafoxanide significantly reduces DLBCL cell survival and activates apoptotic pathways. Furthermore, it inhibits cell division, disrupts mitochondrial function, and increases oxidative stress, all with minimal toxicity to healthy immune cells [36]. At the molecular level, rafoxanide modulates tumor suppressor and oncogenic pathways, including PTEN/PI3K/AKT and JNK/c-Jun cascades, while increasing phosphorylated H2AX levels, thus compromising DNA repair capacity in DLBCL cells [36]. Moreover, in a xenograft mouse model, administration of rafoxanide significantly suppressed tumor volume and increased markers of cell death in neoplastic tissue [36]. Notably, rafoxanide showed dose-dependent cytotoxic effects across six DLBCL cell lines, with IC_50_ values ranging from 19.0 to 37.1 μM, including 19.0 μM for OCI-LY8 and 37.1 μM for DB cells, highlighting its variable potency within this lymphoma subtype [36]. These findings indicate that rafoxanide deserves further research as a possible treatment for DLBCL patients.

## 3. Clinical and Pharmacological Limitations

Rafoxanide is among the drugs currently being repurposed with promising potential for cancer therapy. Recent research suggests that it can effectively influence tumor dynamics through various biological mechanisms, such as inhibiting cell proliferation, inducing apoptosis, modulating autophagy, and boosting immune responses, particularly by activating cytotoxic T lymphocytes.

Although these findings are promising, they are based exclusively on in vitro studies using cancer cell lines and in vivo experiments in animal models. Therefore, further research is needed to evaluate the safety and therapeutic efficacy of rafoxanide in humans, particularly given the complexity of cancer. Moreover, rafoxanide is not approved for human use, with the exception of a single case report describing its administration to a seven-year-old girl with fascioliasis [68]. Of note, niclosamide, another halogenated salicylanilide, is approved by the FDA for the treatment of tapeworm infections and has been administered safely in clinical settings. Numerous studies have demonstrated that niclosamide possesses potent anti-cancer properties, including anti-proliferative and pro-apoptotic effects, along with activity as a STAT3 inhibitor and a negative regulator of Programmed Death-Ligand 1 protein, which may potentiate immunotherapeutic responses [69,70,71,72,73,74]. Moreover, multiple clinical trials have been completed or are ongoing to investigate the potential of niclosamide in cancer therapy, with results to date being mixed—some reporting positive outcomes, while others have shown limited or no efficacy (NCT05188170, NCT02807805) [75,76]. These findings suggest that rafoxanide could be a promising candidate for enhancing tumor cell sensitivity to existing immunotherapies, such as monoclonal antibodies targeting immune checkpoints. Furthermore, potential toxicity toward immune cells appears unlikely, as preliminary observations from our group indicate that rafoxanide does not exert detectable toxicity to human peripheral blood mononuclear cells (personal unpublished data). However, further studies are currently underway to investigate the effects of rafoxanide on different immune cell subsets, as it cannot be ruled out that this compound may influence their viability, cytokine production, or activity.

Overcoming the pharmacokinetic drawbacks of rafoxanide is also crucial for its successful repurposing as an effective anticancer agent. Indeed, its clinical use is constrained by poor oral bioavailability, primarily resulting from extensive first-pass metabolism and limited water solubility. These issues reduce the amount of active drug that reaches systemic circulation, thereby diminishing its capacity to effectively target cancer cells. To improve its clinical potential, strategies should focus on optimizing dosing and drug delivery methods. Alternative administration routes—such as parenteral, transdermal, or nanoparticle-based delivery—can bypass the gastrointestinal tract and reduce metabolic degradation, thereby enhancing systemic exposure. Developing prodrugs or structural analogs with improved pharmacokinetic profiles can enhance stability and bioavailability while preserving anticancer activity. Chemical modifications aimed at enhancing lipophilicity or reducing susceptibility to hepatic metabolism, combined with innovative formulations such as liposomes, micelles, or polymeric nanoparticles, offer additional avenues for optimization. These approaches aim to improve rafoxanide’s pharmacokinetic profile, enhance tumor-specific targeting, minimize off-target effects, and ultimately increase its therapeutic efficacy in cancer treatment.

## 4. Conclusions

The future potential of rafoxanide as a repurposed therapeutic agent will depend on rigorous scientific research, advances in drug delivery methods, and systematic clinical evaluations to validate its efficacy in cancer therapy and beyond. Multi-phase clinical trials will be crucial to identify optimal dosing regimens, assess long-term safety, and determine the patient populations most likely to benefit from this treatment.

## Figures and Tables

**Table 1:** Anti-tumor effects of rafoxanide in different cancer types. Upregulation and downregulation are represented by upward and downward arrows, respectively.

Cancer Type	In Vitro Mechanism of Action	Key Molecular Targets/Pathways	In Vivo Effects	Reference
*Skin cancer*	G1 phase cell cycle arrest	CDK4/6–p-CDK4/6 ↓ cyclin D ↓ Rb/p-Rb ↓	Xenograft nude mice with A375 skin cancer cells: tumor growth ↓	[28]
*Gastric adenocarcinoma*	Cell cycle arrest at the G0/G1 phase	Cyclin D1 ↓ Cyclin E ↓	Xenograft mouse model (SGC-7901 cells): tumor growth ↓ Apoptosis marker ↑ Autophagy marker ↑ Ki67 ↓ p-Akt ↓ LC3B ↑ Autophagic vacuoles ↑	[29]
	Mitochondrial intrinsic apoptotic pathway (loss of MMP)	Cleaved caspase-3 ↑ Cleaved caspase-9 ↑ Release of cytochrome C PARP fragmentation		
	Autophagy ↓	LC3-II ↑ Autophagic vacuoles		
	Cell survival rate ↓	PI3K ↓ p-Akt ↓ p-mTOR ↓		
*Colorectal cancer*	Cell cycle arrest at the G0/G1 phase Endoplasmic reticulum stress (ERS) ↑	p-ERK ↓ Proliferation ↓ CDK4/6 ↓ Cyclin D1 ↓ p-eIF2α (S51) ↑ p-PERK (T981) ↑ CHOP ↑ Ki-67 positive cells ↓	Human CRC explants: Ki-67 positive cells ↓ ATF4 ↑ GRP78 ↑ CHOP ↑ *Apc*^min/+^ mouse model: Tumor score and load ↓ Ki-67 positive cells ↓ p-eIF2α (S51) ↑ cleaved caspase-3 ↑	[30]
	Immunogenic cell death (ICD)	p-eIF2α (S51) ↑ ecto-calreticulin ↑ LC3-II ↑ Extracellular ATP ↑ HMGB1 ↑ Proliferation ↓	Vaccination (syngeneic rafoxanide-treated CT26 cells): tumor score and load ↓	[31]
	TRAIL-induced apoptosis	Apoptosis ↑ Caspase-8 ↑ DR5 ↑ Proteosome degradation c-FLIP ↓ BCL-X_L_ ↓ XIAP ↓ Survivin ↓	Human CRC explants: c-FLIP ↓ Survivin ↓ Xenograft mouse model (CT26 cells): tumor volume ↓ cleaved caspase-3 ↑ c-FLIP ↓ Survivin ↓	[32]
	Antineoplastic effect	p-STAT3 Y705 ↓ p-NF-κB S536 ↓ CDK6/Cyclin D1 ↓	AOM/DSS mouse model: Tumor score and load ↓ Ki-67 positive cells ↓ *Apc*^min/+^ mouse model: p-STAT3 Y705 ↓ p-NF-κB S536 ↓ Organoids: Ki-67 positive cells ↓ p-STAT3 Y705 ↓ p-NF-κB S536 ↓ TILs: p-STAT3 Y705 ↓ p-NF-κB S536 ↓ IL-6 ↓ TNF-α ↓ CD3+ secreting IFN-γ ↑	[33]
*Non-small cell lung cancer*	proliferation ↓ invasion/migration ↓ apoptosis ↑ cell cycle arrest at the G0/G1 phase endoplasmic reticulum stress (ERS) ↑ unfolded protein response (UPR) ↑ Autophagy ↑	Bcl2 ↓ Bax ↑ Cleaved-PARP ↑ Cyclin D1 ↓ Cyclin E ↓ p-Rb ↓ GRP78 ↑ p-eIF2α (S51) ↑ ATF6 (P90) ↓ ATF6 (P50) ↑ Xbp1s LC3 I/II ↑ CHOP ↑	xenograft mouse models (A549 cells): Tumor volume and weight ↓ Ki-67 ↓	[34]
*Multiple Myeloma*	apoptosis (both intrinsic and extrinsic pathways) ↑ cell cycle arrest at the G0/G1 phase: key cell cycle regulators↓ DNA damage response (DDR) signaling ↑	proliferation ↓ caspase-9 ↓ cleaved caspase-3, -8, -9 ↑ Bcl-X_L_ ↓ Bcl-2 ↓ Bax ↑ MMP ↓ cyclin D1, CDK4, CDK6, and cdc25A ↓ p-CHK2 ↑ γ-H2AX ↑ p-p38 MAPK and p-STAT1 ↓ B-Raf V600E ↓	xenograft mouse models (H929 cells): Tumor volume and weight ↓ Ki-67 ↓ Cleaved caspase-3 ↑ TUNEL + γ-H2AX ↑ p-p38 MAPK ↓	[35]
*B-cell Lymphoma*	proliferation ↓ apoptosis ↑ mitochondrial stress response cell cycle arrest at the G0/G1 phase oxidative stress ↑	Caspase-3, -8, -9 ↑ Bcl-2 ↓ Bax ↑ MMP ↓ cyclin D1, CDK4, CDK6, and cdc25A ↓ γ-H2AX ↑ PTEN/p-AKT and p-JNK/p-c-Jun ↑ DNA repair capacity ↓	xenograft mouse model (DLBCL cells): tumor volume ↓ markers of cell death in neoplastic tissue ↑	[36]

Abbreviations: CDK4/6, cyclin-dependent kinase 4/6; Rb, retinoblastoma protein; MMP, mitochondria membrane potential; PARP, Poly(ADP-ribose) polymerase; PI3K, phosphatidylinositol 3-kinase; p-mTOR, phosphorylated mammalian target of rapamycin; p-ERK, phosphorylated extracellular signal-regulated kinase; p-EIF2α, phosphorylated eukaryotic initiation factor 2α; p-PERK, phosphorylated PKR-like endoplasmic reticulum kinase; CHOP, C/EBP homologous protein; ATF6, activating transcription factor 6; GRP78, glucose-regulated protein 78; HMGB1, high mobility group box 1; DR5, death receptor 5; c-FLIP, cellular FLICE-like inhibitory protein; BCL-XL, B-cell lymphoma-extra large; XIAP, X-linked inhibitor of apoptosis; p-STAT3, phosphorylated signal transducer and activator of transcription 3; p-NF-κB, phosphorylated nuclear factor kappa-light-chain-enhancer of activated B cells; DSS, dextran sodium sulfate; AOM, azoxymethane; Apc, adenomatous polyposis coli; IL-6, interleukin-6; TNF-α, tumor necrosis factor-α; IFN-γ, interferon-γ; Bcl-2, B-cell lymphoma/leukemia-2; BAX, Bcl-2-associated X protein; Xbp1s, spliced form of X-box binding protein 1; cdc25A, cell division cycle 25 homolog A; p-CHK2, phosphorylated checkpoint kinase 2; γ-H2AX, γ-H2A histone family member X; p-p38 MAPK, phosphorylated p38 mitogen-activated protein kinase; B-Raf, B-rapidly accelerated fibrosarcoma; TUNEL, terminal deoxynucleotidyl transferase dUTP nick end labeling; PTEN, phosphatase and tensin homolog; p-JNK, phosphorylated Jun N-terminal kinase.

## Data Availability

No new data were created or analyzed in this study. Data sharing is not applicable to this article.
